# Aluminum Reservoir Welding Surface Defect Detection Method Based on Three-Dimensional Vision

**DOI:** 10.3390/s25030664

**Published:** 2025-01-23

**Authors:** Hanjie Huang, Bin Zhou, Songxiao Cao, Tao Song, Zhipeng Xu, Qing Jiang

**Affiliations:** 1 College of Metrology Measurement and Instrument, China Jiliang University, Hangzhou 310018, China; huanghanjie@cjlu.edu.cn (H.H.); caosongxiao@cjlu.edu.cn (S.C.); songtao@cjlu.edu.cn (T.S.); xuzhipeng@cjlu.edu.cn (Z.X.); jiangq2004@163.com (Q.J.); 2 State Key Laboratory of Fluid Power and Mechatronic Systems, Zhejiang University, Hangzhou 310027, China

**Keywords:** 3D vision, welding defects detection, laser scanning, plane correction

## Abstract

Welding is an important process in the production of aluminum reservoirs for motor vehicles. The welding quality affects product performance. However, rapid and accurate detection of weld surface defects remains a huge challenge in the field of industrial automation. To address this problem, we proposed a 3D vision-based aluminum reservoir welding surface defect detection method. First of all, a scanning system based on laser line scanning camera was constructed to acquire the point cloud data of weld seams on the aluminum reservoir surface. Next, a planar correction algorithm was used to adjust the slope of the contour line according to the slope of the contour line in order to minimize the effect of systematic disturbances when acquiring weld data. Then, the surface features of the weld, including curvature and normal vector direction, were extracted to extract holes, craters, and undercut defects. For better extraction of the defect, a double-aligned template matching method was used to ensure comprehensive extraction and measurement of defect areas. Finally, the detected defects were categorized according to their morphology. Experimental results show that the proposed method using 3D laser scanning data can detect and classify typical welding defects with an accuracy of more than 97.1%. Furthermore, different types of defects, including holes, undercuts, and craters, can also be accurately detected with precision 98.9%.

## 1. Introduction

The reservoir dryer is a key component of a vehicle’s heat exchange system and is primarily responsible for filtering liquids and contaminants from the air entering the cooling system. This prevents damage to the compressor and other components. To ensure that the reservoir dryer functions properly, welding is often used during assembly to ensure that it is hermetically sealed. However, external disturbances during the welding process, such as mechanical vibration, inconsistent product placement, and variations in welding parameters, can cause defects such as holes, undercuts, and craters in the weld [1]. These defects can negatively affect the performance of the component, so defect detection is essential to reduce production costs, optimize the process, and ensure quality.

**Figure 1:**
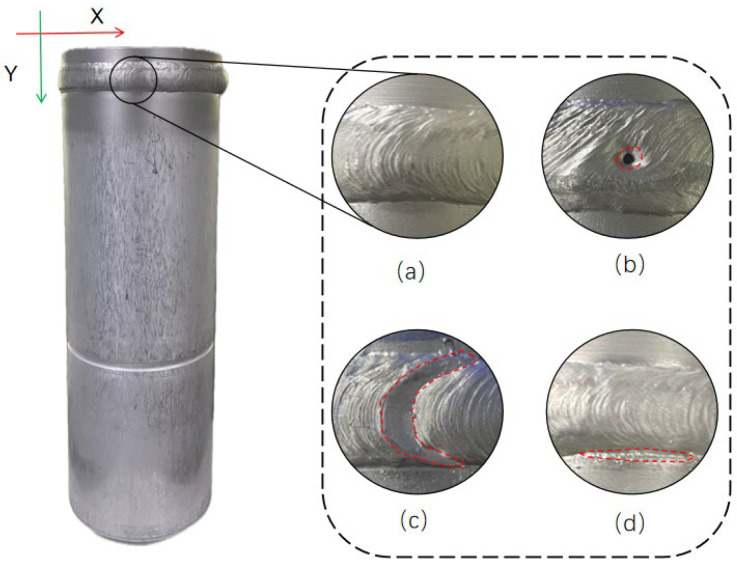
Reservoirs and welds with different types of defects. Defects are circled in red: (**a**) normal weld; (**b**) hole defects; (**c**) arc crater defects; (**d**) undercut defects.

Figure 1 illustrates the morphological characteristics of these three defects observed in the weld: (a) is a defect-free weld, (b) is a hole defect characterized by a circular or elliptical cavity in the weld, (c) is a crater defect characterized by the height of the area being significantly lower than the rest of the weld, and (d) is an undercut defect characterized by the fact that it is at the edge of the weld, lower than the base metal area.

Depending on the welding technique, there are several methods available for detecting welding defects. For argon arc welding, common inspection methods include magnetic particle inspection, ultrasonic inspection, eddy current inspection, penetration inspection, magneto-optical imaging inspection, infrared inspection, and machine vision inspection. Each method is based on a different principle and has a specific range of inspection equipment and applications. For example, eddy current inspection [2] relies on electromagnetic induction to detect defects by predicting material thickness. It has the advantages of rapid detection and non-contact measurement. Infrared inspection [3] is a non-destructive method that identifies defects by measuring infrared radiation and inferring surface temperature distribution. These methods, although highly accurate, are often costly and difficult to automate.

In recent years, machine vision-based image processing and point cloud processing have gained popularity in the field of defect detection due to their cost-effectiveness and ease of deployment. Early applications based on 2D image processing laid the foundation for welding defect detection. For example, Burch, S. F [4] proposed an image processing-based weld image processing system that allows for real-time monitoring and analysis of inspection results. However, those methods had limitations in defect extraction. Subsequent studies have introduced improved methods, such as extracting texture features from gray-scale co-occurrence matrices and two-dimensional Gaussian functions [5], which have improved the classification accuracy. In addition, methods based on fuzzy set theory and low-pass filtering have been widely used to improve defect detection algorithms. J Sun et al. [6] proposed a modified background subtraction method based on a Gaussian mixture model to extract the feature regions of weld defects. Then, they designed a weld detection and classification algorithm based on the extracted features. Despite these advances, 2D image processing still has difficulties in accuracy due to its susceptibility to lighting conditions, thus limiting its wide application.

Unlike 2D imaging, 3D point cloud imaging is less affected by surface illumination and contains height information, thus improving the accuracy of defect detection on complex surfaces. Point cloud-based defect detection methods include contour line-based detection, template matching, and local surface characterization [7]. In contour line-based studies, methods such as alignment and point cloud contour fitting are common. For example, W Huang [8] et al. used contour line information to detect weld quality. Dejin Zhang et al. [9] combined candidate points with a minimum cost spanning tree algorithm, which can be used to detect crack information and categorize pavement defects based on the deformation depth. In addition, Qinbing Zheng et al. [10] detected the quality of additively manufactured parts whose principle is also based on the analysis of contour lines.

Methods based on localized surface features rely on geometric and topological information. For example, Delin Huang et al. [11] used wavelet entropy to segment and analyze the surface region of an engine piston to detect defects. In addition, surface feature-based defect extraction methods have been used for the detection of damage on cabbages [12], airplane surface depressions [13], railroad track defects [14], and concrete surface damage [15]. However, the object surface texture pair information poses a challenge to this detection method; for this reason, ET Lee et al. [16] converted the 3D point cloud data into surface normal vectors, and then processed them using Gabor filters, which can effectively reduce the interference in detecting regular surface defects.

The advent of deep learning has further advanced welding defect detection, with models such as convolutional neural networks excelling in image recognition and classification. By training on large datasets, these models can efficiently detect defects and handle complex and diverse situations. For example, L Chen et al. [17] used machine learning techniques combined with filtering, segmentation, and point clustering, achieving high accuracy in surface defect detection. Diyu Guan et al. [18] have proposed an automatic weld feature recognition algorithm based on an improved U-Net neural network. The experimental results demonstrate that by extracting the global features of the weld image through the U-Net network and fusing multi-scale laser fringe information, the accuracy can reach 99.17%. David Curiel et al. [19] mounted a laser profiler on a robot and reconstructed the weld in three-dimensional space. Signal processing was used to remove noisy data. A classification algorithm combining signal processing and artificial intelligence was used to accurately classify the weld. However, deep learning models require large quantities of labeled data, which can be difficult to obtain. In addition, their “black box” nature raises concerns about transparency and trust in industrial applications.

Challenges remain in using machine vision for weld defect detection, especially for 2D imaging. The inconsistency of the weld seam and the influence of ambient light sources can affect the robustness of the algorithms. Current studies on laser line-based weld defect detection typically focus on a single contour line approach that ignores adjacent point clouds, resulting in high spatial dispersion of weld edges. In addition, during the inspection process, the weld image can be distorted due to mechanical vibrations or changes in the pitch and yaw angles of the container, affecting subsequent steps. To address these issues, we employ a plane correction technique that leverages the slope of the plane region to enhance the accuracy of the scanning process and facilitate the detection of defects. Subsequently, we utilize the information from the adjacent point cloud and a methodology based on the surface characteristics of the point cloud to achieve the detection of defects.

In summary, the main contributions of this paper are as follows:

1. The slope-based plane correction method is used to preprocess 3D point cloud data to solve the distortion caused by mechanical vibration and eccentric rotation of the reservoir during the detection process.
2. In order to effectively extract welding defects, a defect detection method based on point cloud surface features is used. The extraction results are classified using a method based on the characteristics of weld defects, which achieves an accurate distinction between defect types.
3. By using a method that combines surface feature extraction with two-stage registration extraction, the extraction of large crater defects is achieved, thereby improving the accuracy of defect area extraction.

The structure of this paper is as follows: Section 2 describes the configuration of the inspection system, and Section 3 serves as an introduction to the inspection methods, including plane correction, defect extraction base on surface features, and differential template, and classifies the convinced extraction results according to the extracted point cloud features. Section 4 outlines the related experiments and Section 5 provides the corresponding conclusions.

## 2. Vision System Design

**Figure 2:**
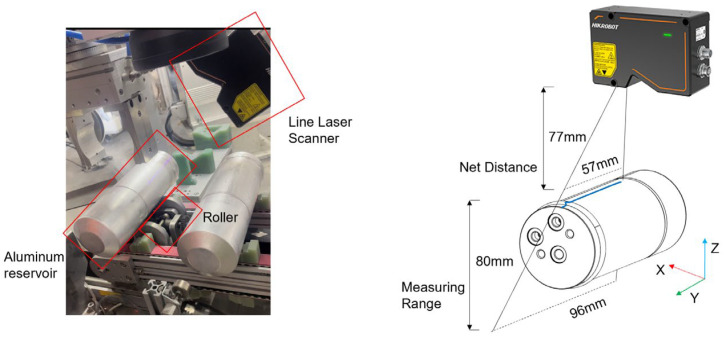
Weld inspection system.

This study focuses on acquiring extensive 3D point cloud data from the surface of an aluminum reservoir during production. The defect detection system, along with the point cloud data acquisition device, is illustrated in Figure 2. The detection system can be divided into three parts: PLC, executive mechanism, and vision system. During detection, the PLC controls the executive mechanism to move the reservoir to the designated position and controls the motor to drive the rollers at the bottom of the reservoir to rotate at a constant speed. At the same time, the PLC sends a detection signal to the visual system, which controls the line laser profiler to scan the product at a fixed frequency and output the detection result. The PLC controls the executive mechanism to unload the product according to the detection result.

Specifically, this system uses the Hikvision MV-DP2307-10H Line Laser Profiler, produced by Hikvision, a company based in Hangzhou, China, as the point cloud acquisition device. It calculates the three-dimensional contour of the object’s surface using triangulation by emitting a laser line and capturing the reflected image with a camera. Compared to a 2D camera, no additional light source is needed, the laser emitted by the line laser profiler itself is characterized by high brightness, and with the filter mounted on the receiver, it is able to isolate the external light, so the external light on the system will not have an impact.

The point cloud data are organized within a coordinate system defined during the scanner’s acquisition process. In this system, the origin corresponds to the first point captured by the scanner. The *X*-axis aligns with the laser line emitted by the scanner, while the *Y*-axis follows the scanner’s motion path. The *Z*-axis represents the height information, indicating the surface height being scanned. In order to meet the needs of the field inspection beat, after experimental adjustment, the image acquisition frame rate is set to 2000 frames per second, according to a reservoir diameter of 75 mm, a roll angular speed of 180 degrees per second can be known as the resolution of the Y direction for the 0.06 mm/point; the X direction resolution is also set to 0.06 mm/point, and the resolution in the Z direction is 0.005 mm/point. This coordinate system is crucial for accurately mapping and analyzing the receptacle’s surface for defects during production.

## 3. Methods

### 3.1. Overview

**Figure 3:**
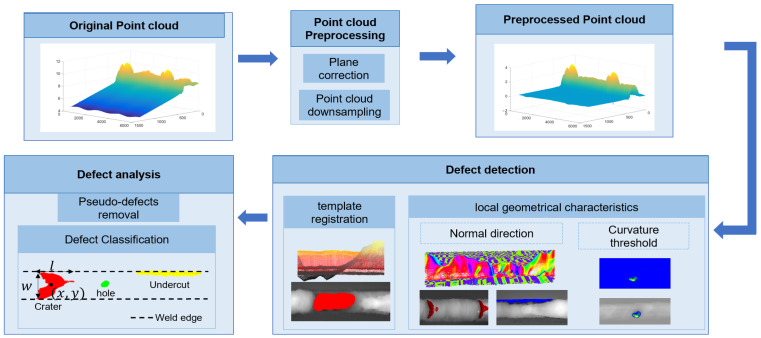
Flow of defect detection method.

The detection framework, as illustrated in Figure 3, involves three key steps:

In the first step, cloud data preprocessing, the point cloud data acquired by the line laser profiler are corrected by plane correction and point cloud downsampling to restore the curved weld to the ideal state and to reduce the quantity of data in order to increase the inspection speed.

In the second step, surface defect detection, a detection method based on point cloud surface features was explored, and the curvature and normal vector directions were designed according to the weld defect features; a two-stage alignment method was used to extract the portion of the crater defects that were difficult to extract from the features.

In the third step, the extracted defects are removed as pseudo-defects and the extraction results are categorized according to the type of defect.

### 3.2. Point Cloud Preprocessing

#### 3.2.1. Plane Correction

**Figure 4:**
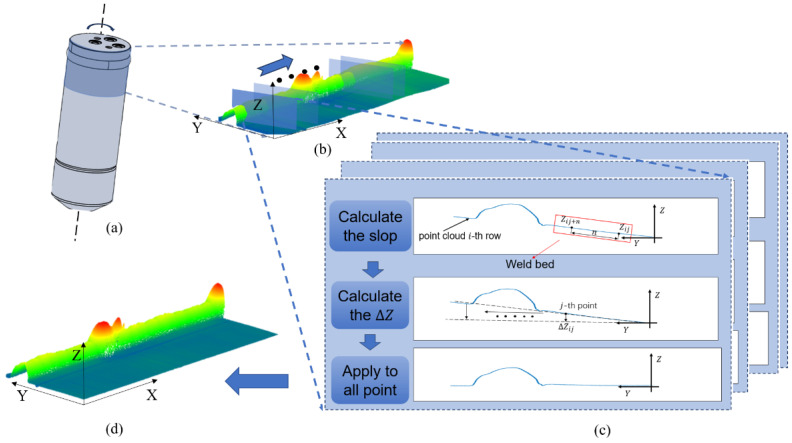
Planar correction process: (**a**) eccentric rotation of the reservoir; (**b**) pre-correction point cloud; (**c**) correction step; (**d**) post-correction point cloud.

During the process of data acquisition, the tilt and distortion of the collected data may be attributed to several factors, including the angle at which the sensor is erected, mechanical vibration of the platform, and non-centering of the rollers. To address this issue, the collected data are typically subjected to plane correction. For plane correction, Random Sample Consensus (RANSAC) is a widely used method for fitting planes, particularly effective when correcting angles between the camera and the subject being photographed [20]. However, RANSAC is less effective in addressing distortions caused by eccentric rotation of the product, as illustrated in Figure 4a,b. To address this issue, this paper employs a slope-based correction method tailored to the characteristic that the surface of the point cloud is a ruled surface.

In this approach, the slope of each point cloud row is first calculated based on the reservoir’s planar region. Subsequently, the slope is adjusted to zero by updating each point within the row, thereby ensuring that all rows are level. Finally, the height of the plane region within the point cloud is used to align each row to a uniform reference plane. This method effectively corrects the distortion resulting from eccentric rotation, leading to more accurate defect detection in the welds.

In first step, the point cloud slope of each line is obtained, and for the weld point cloud data, all the contour lines in the YZ plane are extracted along the X direction; for the extracted contour lines, as in Figure 4c, the planar region corresponding to the reservoir for the contour line is selected, and two points with a distance of n are selected in the region; the slope of the region is calculated according to Equation (1):

(1) $$k_{i}=\frac{Z_{ij}-Z_{ij+n}}{n}$$

where i,j,n denotes the number of rows and columns in the current point cloud row and the distance between the two selected points along the *y*-axis, and $Z_{ij}$ denotes the height of the *z*-axis of the selected point cloud.

When pre-segmenting the packaging point cloud, the overall packaging point cloud volume size and direction are determined based on the minimum bounding box. In the second step, each point on the current point cloud row is updated according to the slope of the current point cloud row, because the heights of the points along the y direction can be considered as an arithmetic progression sequence, so the difference between every neighboring point can be approximated as the slope $k_{i}$. Each point on the row is updated by the following equation:

(2) $$\left\{\begin{array}{l} Z_{ij}^{\prime }=Z_{ij}-\Delta Z_{ij}\\ \Delta Z_{ij}=k_{i}\left(j-1\right) \end{array}\right.$$

At each point on the contour line, update the height of the point according to the slope of the contour line (set the first point in the current row as the datum). Due to the characteristics of the laser scanner, the distances between two neighboring points on the contour line are equal, so for the *j*-th point in *i*-th row, the difference in z direction between the value of the point and the datum is $k_{i}\left(j-1\right)$, and subtracting this value changes the point to the same height as the datum. In this way, the slope of the contour line in this row is 0, and this step is repeated for all contour lines.

Finally, set the datum plane at height 0. For each row of contour lines, calculate the height difference between the first point on that contour line $Z_{ij}^{\prime }$ and the datum plane, and add all points on that contour line to that height difference. Repeat this step for all contour lines. Achieve height uniformity for all contour lines.

By this operation, the tilt correction and bending correction of the point cloud are completed. The correction effect is shown in Figure 4d.

#### 3.2.2. Point Cloud Downsampling

Point cloud data obtained from line laser scanning are typically characterized by high density. To reduce computational costs while preserving the original features of the point cloud, it is essential to downsample the data. Various point cloud downsampling methods exist, including voxel grid sampling [21], random sampling [22], and curvature-based sampling [23], each suited to different applications. In voxel grid sampling, the size of the voxel determines the sparsity of the point cloud, and the uniform distribution of sampled points after downsampling makes it well suited for this system. Preprocessing the point cloud data to eliminate irrelevant information enhances the efficiency of subsequent defect detection processes.

### 3.3. Surface Defect Detection

For welding defects, the point cloud data have the following characteristics: the height of the defective area, the *z*-axis direction, changes drastically and its normal vector is oriented in a specific direction depending on the defect type; relatively, the height of the normal weld *z*-axis varies gently, and the direction of the point cloud is oriented in a fixed direction. Therefore, a method based on point cloud surface features is used to segment the defective region.

Defect detection methods based on point cloud surface features rely on local surface information, including curvature, normal vector, geometric, and topological data. These methods divide points or sub-regions into larger regions based on the consistency metric of local surface features. In geometric features, the normal vector and curvature of the point cloud can produce effective feature differences. Even after the point cloud has undergone denoising and smoothing, it can still be detected effectively.

The vector of the point cloud represents the orientation information of each point surface, whereas the surface curvature describes the degree of variation of the point cloud through the eigenvalues. These features provide information about the local surface orientation and shape, which is crucial in the analysis of the geometric features of the point cloud, including segmentation, classification, and feature detection.

**Figure 5:**
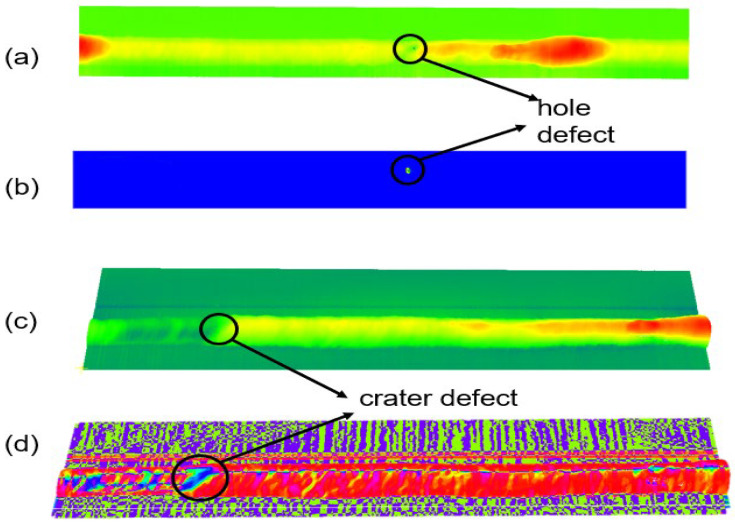
Defect depth map and curvature and normal vector scalar map: (**a**) elevation map of hole defects; (**b**) curvature map of hole defects; (**c**) elevation map of arc crater defects; (**d**) normal vector direction map of curvature defects.

Figure 5a,c show the scanned point clouds of hole defect and crater defect welds, from their corresponding curvature Figure 5b, normal vector direction Figure 5d. From which it can be seen that the defects extracted using the surface features are significantly different from the other regions, so different feature sampling for different defects is a feasible extraction method.

Typically, these surface properties can be obtained by characterizing the covariance matrix of the local neighborhood. For each point $p_{i}=\left(x_{i},y_{i},z_{i}\right)$, the covariance matrix of the point can be expressed as follows:

(3) $$\left\{\begin{array}{c} C=\frac{1}{N}\sum_{i=1}^{N}\left(p_{i}-\bar{p}\right)\cdot {\left(p_{i}-\bar{p}\right)}^{T}\\ \bar{p_{i}}=\frac{1}{N}\sum_{j=1}^{N}p_{ij} \end{array}\right.$$

where N is the set of neighboring points for point $p_{i}$ with radius r and $\bar{p}$ is the neighborhood point; the eigenvalue decomposition of the covariance matrix C is performed using Equation (4) to find the eigenvalue $\lambda_{1},\lambda_{2},\lambda_{3}$ and its corresponding eigenvector $v_{1},v_{2},v_{3}$:

(4) $$Cv_{j}=\lambda_{j}v_{j}$$

where $\lambda_{1}<\lambda_{2}<\lambda_{3}$, and the normal vector $\vec{n}$ is usually defined as the eigenvector of the smallest eigenvalue pair of the covariance matrix C. The eigenvectors of the covariance matrix correspond to the normal vectors of the point, where the eigenvector of the smallest eigenvalue is the normal vector of the point.

The surface curvature is calculated by Equation (5), where the curvature $\sigma_{p}$ is obtained by estimation from the eigenvalues of the covariance matrix:

(5) $$\sigma_{p}=\frac{\lambda_{1}}{\lambda_{1}+\lambda_{2}+\lambda_{3}}$$

#### 3.3.1. Algorithm for Hole Detection Base on Curvature

Surface defects of welds typically manifest as holes, undercuts, and craters. Among these defects, holes are among the most prevalent. Figure 5a illustrates the scanning results of a weld exhibiting hole defects on the surface. These defects are typically characterized by the emergence of pits or voids on the surface of the weld. Consequently, the alteration in surface characteristics is more discernible in the region of the defect than in other areas.

**Figure 6:**
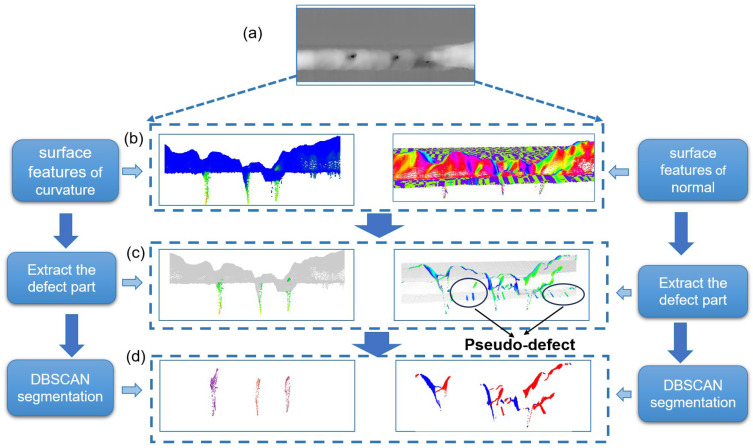
Weld nibbling region profile with the direction of the normal vector in the region: (**a**) point cloud depth map; (**b**) curvature and normal vector direction map; (**c**) curvature and normal vector direction extraction results; (**d**) point cloud clustering results.

The surface curvature of the hole defect region is highly variable and possesses a higher degree of curvature than that observed in the smooth surface of a normal weld. Based on the aforementioned geometric analysis, a surface curvature-based algorithm was employed for the detection and characterization of the defects. As illustrated in Figure 6b, the curvature of the defective region is markedly elevated in comparison to that of the typical region of the weld. Furthermore, the closer the region is to the center of the hole defect, the higher the curvature. Consequently, the damage region can be extracted by establishing the neighboring points N and the curvature threshold $\delta_{1}$, utilizing varying curvatures. The curvature parameter of the feature points is employed as one of the threshold values for the extraction of the final point cloud, which is deemed to be the correct damage region. The results of the extraction are illustrated in Figure 6c, which depicts the extracted defective region point cloud. The method demonstrates effective extraction of the hole defective region. Subsequently, the DBSCAN (Density-Based Spatial Clustering of Applications with Noise) algorithm is employed to perform a clustering operation on the aforementioned threshold-segmented point cloud. The clustering result is illustrated in Figure 6d.

During the detection process, it is inevitable that some overlap will occur between the arc starting and arc closing regions of the weld due to the inherent characteristics of the welding process. Additionally, the surface of the weld itself is rough and uneven, which results in a height difference between this region and other regions. This height difference may lead to the false detection of the normal region, as the curvature of this region increases in comparison to other regions. To address this issue, a secondary screening of the regions extracted through curvature threshold segmentation is employed. The subsequent sections provide a detailed description of this operation.

#### 3.3.2. Algorithm for Crater and Bite Edge Detection Based on Normal Vector Direction

Arc crater defects exhibit distinct geometrical characteristics when compared to hole and undercut defects. Figure 6c illustrates the scanning results of a weld exhibiting arc crater defects on the surface. Arc crater defects typically exhibit a gradual transition in comparison to the surrounding normal region of the weld, resulting in a minimal discrepancy in surface curvature between the defect and the remaining weld regions. This makes it challenging to extract the defect’s curvature efficiently through direct calculation. Instead, an angle is formed between the defective region and the normal weld. To address this challenge, we employ a normal direction-based algorithm to detect and characterize the defects using the aforementioned geometrical analysis. The normal vector direction of the region is calculated, and the point cloud corresponding to the normal vector of this direction is extracted. This is achieved by transforming the normal vector, designated as $\vec{n}$, for each point within the point cloud into an angular scalar.

**Figure 7:**
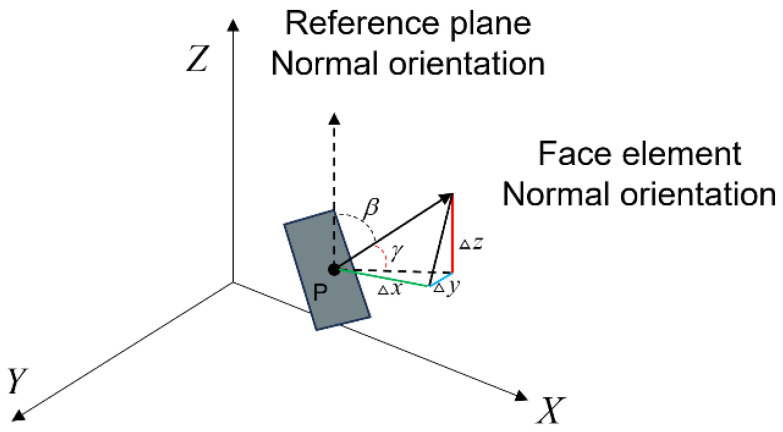
Principle of normal vector direction calculation.

As illustrated in Figure 7, point P represents a specific point within the point cloud. The angle β represents the discrepancy between the vector and the normal vector of the surface of point P. Similarly, the angle γ denotes the angle between the horizontal reference plane and the normal vector of the surface of point P. The value of β can be calculated using the following Equation:

(6) $$\gamma =\tan^{-1}\left(\frac{\Delta z}{\sqrt{\Delta x^{2}+\Delta y^{2}}}\right)$$

where x, y, and z are the differences between two vertices of the surface normal vector on each axis in the 3D coordinate system, and ${tan}^{-1}$ is the arctangent function. Therefore, the angle β is equal to (90−γ).

Figure 5d shows the vector plot of normal vector directions corresponding to the calculated arc crater defective weld, in which different normal vector directions are shown by different colors, and the normal vector directions are 0° to 360°. As illustrated in the normal vector direction map, the normal vector direction of the point cloud situated at the beginning and end of the arc crater defect is markedly disparate from that of the normal region. As illustrated in Figure 6b,c, the arc surface region of the crater defect can be delineated by setting the angle interval. It has been demonstrated that the optimal result for the defective region is obtained when the angle threshold is set to a range of 270° to 320°. Furthermore, the presence of undulations on the surface of the weld necessitates the extraction of this region when defects are identified. To address this, a larger minimum number of points in the DBSCAN clustering is employed to filter out the affected region.

**Figure 8:**
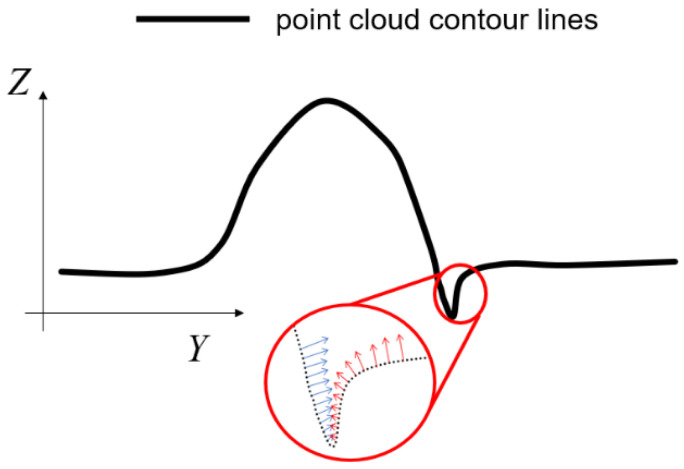
Contour lines of undercut defects with the direction of the normal vector of the region.

In the case of undercut defects, a downwardly depressed region is observed at the intersection of the weld and the base metal. This region is usually distributed in a band. In consequence of this characteristic, the direction of the normal vector in the depressed area can be observed. The arch shape of the weld cross-section determines the range of normal vector directions for each of the two arcs extending from the apex of the arch to the base metal area. In the undercut region, as illustrated in Figure 8, the contour line of the defective region exhibits a V-shaped downward depression, resulting in disparate normal vector directions on either side of the V-shaped defect. The normal vector direction of the left side of the V-shape is identical to that of the arch region connected to it, whereas the right side of the V-shape exhibits the opposite direction.

Consequently, the highest point of the weld seam is taken as the center of division, with the weld seam divided into two regions along the *Y*-axis. In order to obtain the point cloud of undercut defects, the point clouds corresponding to the normal vectors in specific directions are extracted for these two regions, respectively.

#### 3.3.3. Defect Detection Algorithm Based on Template Difference

Although the curvature and normal vector direction can be used to quickly locate the position of the defects, the extraction of the defective region, especially the crater region, is not complete. In order to be able to realize the complete extraction of the defective region to analyze the defects, we use a double-aligned template matching method for further extraction.

Initial matching is performed by extracting local descriptors or overall shapes on the point cloud of the standard point cloud and the point cloud to be measured.

We use the overlapping region of starting and closing arcs formed during the welding process as the feature region and gray-scale images for fast template matching. The specific steps are as follows:

1. Obtain the characteristics of the starting arcs by means of a feature pyramid.
2. Matching is performed on each gray-scaled image to obtain the matching center (x,y).
3. Create a rectangular ROI region (x,y,l,w) of size as a mask, where l and w are the length and width of the rectangular region, which are determined by the length and width of the weld seam. Apply this ROI mask to the target point cloud so that only the ROI region is retained to complete weld seam localization and extract the weld seam.

This step allows for the approximate position of the weld to be identified and the weld to be cut to a uniform length. Subsequently, the ICP (Iterative Closest Point) algorithm [24] is employed to achieve precise alignment of the two point cloud models. In order to achieve the requisite alignment, it is necessary to utilize a template, which is typically the CAD (computer-aided design) model of the measured work-piece [25], but since the weld seam is not a standard work-piece, it is impossible to obtain a standard CAD model; for this reason, we use multiple filtered weld seam point clouds, which are used as the standard template after the alignment, downsampling, and shifting least squares smoothing operations.

**Figure 9:**
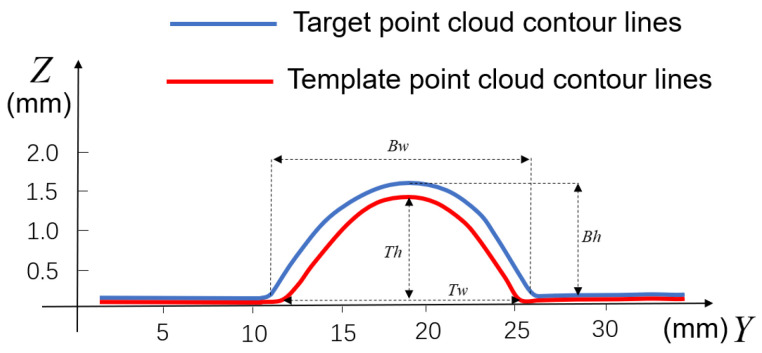
Weld contour lines and their widths and heights for target and template point clouds.

Since it is not possible to have a perfect match between the fabricated standard template and the target weld, especially because the width and height of the weld are not exactly the same in different weld seams, the standard template needs to be adjusted to the dimensions of the target weld in order to minimize the subsequent fitting error. As shown in Figure 9, we achieve this by defining the scaling matrix S with the scaling factor Ry,Rz, which is as in Equations (7) and (8):

(7) $$S=\left[\begin{array}{ccc} 1 & 0 & 0\\ 0 & Ry & 0\\ 0 & 0 & Rz \end{array}\right]$$

(8) $$\left\{\begin{array}{l} Ry=\frac{Tw}{Bw}\\ Rz=\frac{Th}{Bh} \end{array}\right.$$

Among them, Ry,Ty,Bw is the Y direction scaling parameter of the point cloud, the width of the target weld, and the template weld, and Rz,Th,Bh is the Z direction scaling parameter of the point cloud, the height of the target weld, and the template weld, respectively. Each point of the point cloud of the template weld is subjected to matrix multiplication with the scaling transformation matrix to obtain the transformed point cloud, so that the template weld and the target weld are consistent in the direction of width and height, which can further minimize the error.

After that, the weld and template are aligned using the ICP method. The core idea of the ICP algorithm is to find the nearest point between the target point cloud and the template point cloud by iterative means under certain constraints ($p_{i},q_{i}$), and then calculate the optimal rotational translation matrix (R, t), such that the squared error function is minimized, where the squared error function E is shown in Equation (9):

(9) $$E=\frac{1}{m}\sum_{i=1}^{m}{\left\|q_{i}-\left(Rp_{i}+t\right)\right\|}^{2}$$

where m is the number of nearest point pairs, $p_{i}$ is a point in the target point cloud, $q_{i}$ is the nearest point corresponding to $p_{i}$ in the source point cloud, R is the rotation matrix, and t is the translation vector.

**Figure 10:**
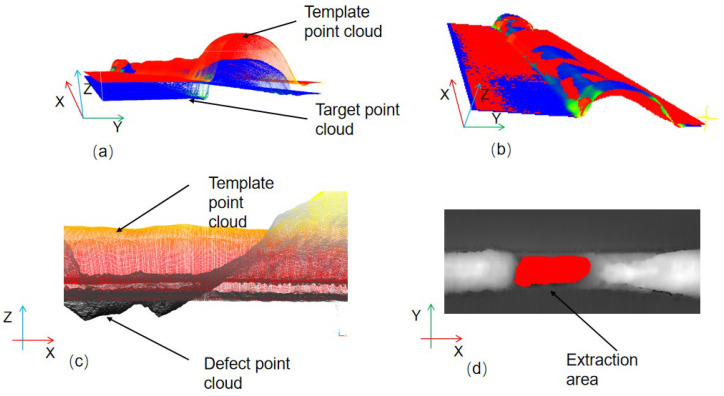
Differential thin plate detection process: (**a**) pre-alignment template with target point cloud; (**b**) post-alignment template with target point cloud; (**c**) post-alignment side view of defective region; (**d**) defective region extraction results.

Figure 10a,b illustrate a comparison of the welds before and after registration. Figure 10c illustrates the alignment outcomes of the defective region, which can be further delineated by calculating the distance between the aligned template and the target weld. Subsequently, a k-d tree calculation is conducted on both point clouds following alignment, thereby facilitating the expeditious identification of neighboring points. The nearest template point cloud to each point of the target point cloud is identified using a k-d tree, and the distance between the two *Z*-axis heights is calculated. The height threshold serves to distinguish between points that are deemed to be defective and those that are not. Points whose height difference exceeds the threshold are extracted and clustered, thereby enabling the segmentation of the candidate region and the subsequent extraction of the defective region. The extraction effect is illustrated in Figure 10d.

### 3.4. Pseudo-Defect Removal and Defect Classification

According to the above steps, potential defective areas with high curvature and specific angles are obtained. However, due to the inherent surface roughness of the weld, normal regions may be mistakenly recognized as defects. In this regard, we use a threshold filtering method, which is used to eliminate pseudo defects and ensure the accuracy and reliability of detection. We use a defect feature matrix on the clustered point cloud data to determine whether the defect is a pseudo-defect. The defect feature factors are as follows:

(10) $$Q=\left[x_{i},y_{i},l_{i},w_{i},h_{i}\right]$$

where $x_{i},y_{i}$ is the center coordinate value of the point cloud ensemble bounding box, $l_{i},w_{i}$ is the length and width of the ensemble bounding box, and $h_{i}$ is the variance of the depth values of all points in the ensemble. The term i (i=1,2…n) represents the number of the extracted area. This information is obtained based on the MVBB (Minimum Volume of Bracketed Boxes) information of the point cloud after clustering.

The judgment process is divided into two parts: (1) determine whether the set is defective; (2) if the set is defective, classify the set according to the type of defect.

The following constraints should be satisfied to determine whether the current point set is a defect:

(11) $$\left\{\begin{array}{c} \delta_{2}<y_{i}<\delta_{3}\\ h_{i}<\delta_{4} \end{array}\right.$$

Among them, $\delta_{2},\delta_{3}$ is the error threshold in the y direction of the geometric center, respectively, the value of the left and right edges of the weld, and the results beyond the edges of the weld are considered as pseudo-defects. In order to prevent the influence of noise on the calculation of height, $\delta_{4}$ is set as the variance threshold of the height of the points in the set, and according to the characteristic that the height falloff of the defective region is larger than that of other regions, the result with smaller height falloff is considered to be a pseudo-defect.

During the welding process, defects can occur on the surface of the weld due to various factors such as welding parameters, trajectory movement, and heat effects. After extensive analysis and observation of the weld surface, the defects are primarily categorized into three types: holes, craters, and undercuts. These defects share some common characteristics: the boundaries of the affected regions are often irregular, and the midpoint of the defect region is typically lower in height than the surrounding normal regions.

However, these defects also differ in specific aspects such as length, width, and the location of the defect’s center. For example, undercuts are generally narrower and longer than holes or craters and are exclusively found along the edges of the weld.

To effectively classify these defects, the following methodology is proposed:

1. Craters: These are identified when the width of the defect is close to the width of the weld seam, and the center of the defect is aligned with the center of the weld seam. If these criteria are met, the defect is classified as a crater.
2. Holes and undercuts:

When the defect width is less than that of the weld seam, further classification is based on the location of the defect center and the aspect ratio (length-to-width ratio) of the defect. If the defect center is at the edge of the weld seam and the aspect ratio is greater than 2, the defect is classified as an undercut. If the defect center is between the left and right edges of the weld seam and the aspect ratio is less than 2, the defect is classified as a hole.

This method allows for the accurate differentiation of defect types based on their geometric properties and locations relative to the weld seam. In the above process, the surface characteristics of all defects were characterized. 

## 4. Experiments and Analysis

### 4.1. Experimental Preparation

The experimental system consists of line laser camera, industrial computer, programmable logic controller (PLC), and other necessary mechanical components. The algorithm is implemented in Python 3.10. In the experiment, the size of the reservoir is a cylinder of ⊘75 mm × 200 mm, and the length of the weld seam after welding is 240 mm, the width is around 9 mm to 12 mm, and the height is 1.5 mm. Different objects are downsampled using different voxel sizes; voxels that are too large reduce measurement accuracy, and voxels that are too small slow down the computation process, thus reducing detection speed. In order to balance the measurement accuracy and algorithm efficiency, the voxel size was determined to be 2 based on several experiments. Once the voxel size is determined, the number of points on the surface of the object can be roughly calculated according to the size of the object, and then the bounding box threshold is determined according to the principle of the point cloud segmentation algorithm. The parameter $\delta_{2},\delta_{3}$ is set based on the size of the actual weld, and the difference threshold of the height square $\delta_{4}$ is set to 0.4 by comparing the defective region with the pseudo-defects.

### 4.2. Planar Correction Experiment

**Figure 11:**
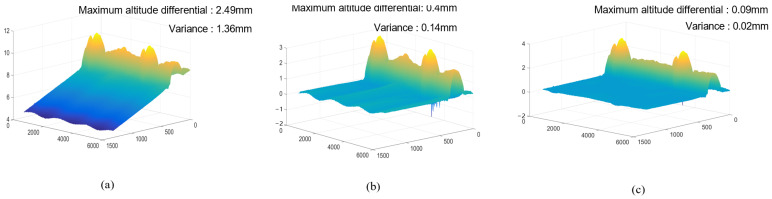
Correction effect of different methods on the point cloud: (**a**) pre-calibration point cloud; (**b**) calibration results using the RANSAC method; (**c**) calibration results using our method.

We used the Gaussian variance of the maximum height difference and height difference in the planar region to verify the effect of planar correction. A comparison of using RANSAC with the present method is shown in Figure 11, where (a) is the weld before correction, (b) is the result of correction using the RANSAC method, and (c) is the result of correction by the present method.

Before correction, the height difference and variance of the welded joint cloud were 2.49 mm and 1.36 mm, respectively. As can be seen from Figure 11a, the plane is not only substantially tilted, but also significantly distorted at the plane.

For comparison, we used the RANSAC method for plane correction; as can be seen from Figure 11b, the maximum height difference and variance were reduced to 0.4 mm and 0.14 mm, respectively, though. However, the distortion phenomenon on the weld surface is not improved.

Comparing with the results of RANSAC, in the results corrected by our method, the maximum height difference and variance are 0.09 mm and 0.02 mm, respectively, and it can be seen from Figure 11c that the distortion phenomenon on the weld surface basically disappeared, which indicates that the effect of surface thinning by the slope method is better than that of the RASNSAC method, and the weld characteristics can be better restored.

### 4.3. Defect Extraction Experiment

In the context of curvature-based threshold segmentation on point cloud data, two key parameters warrant particular attention: the neighborhood radius of curvature and the curvature threshold. The domain radius, as a crucial parameter for regulating the curvature, influences the computational stability. If the neighborhood radius is insufficiently large, it may encompass an inadequate number of neighborhood points, resulting in noise-sensitive computational outcomes and an unstable curvature estimation. If the neighborhood radius is set to a value that is too large, an excessive number of irrelevant points may be introduced, which will result in the local geometric features losing their detailed characteristics due to excessive smoothing. It is therefore essential to select an appropriate neighborhood radius in order to achieve an optimal balance between computational stability and accuracy. The domain radius is set to 10, 15, 20, and 25, respectively. In addition, the defect extraction curvature threshold is set to 0.0005, 0.001, and 0.002, respectively.

**Figure 12:**
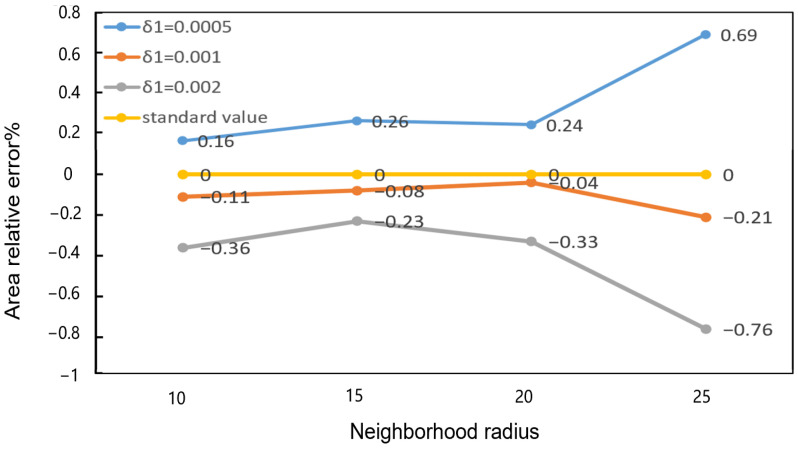
Relative errors of defect extraction with different neighborhood radius and curvature thresholds.

Five groups of point clouds with varying hole sizes were selected to form the defect data for experimentation. The ratios of extracted area to measured area were calculated for varying parameters. The results are shown in Figure 12, where the horizontal coordinate represents the radius of curvature neighborhood, while the vertical coordinate depicts the relative error between the extracted defect area and the actual measured area with different curvature thresholds and neighborhood radii. A value closer to the standard value in the horizontal coordinate indicates a superior extraction effect.

As indicated by the ratio of the extracted area to the actual defect area, the combination with a neighborhood radius of 20 and a curvature threshold of 0.001 is the most closely aligned with the standard value. Consequently, this combination of parameters is deemed to be a reasonable and optimal choice.

The selection of an appropriate angle threshold is of paramount importance in the process of extracting defects by normal vector direction, as it directly affects the accuracy of the extraction. In the experimental procedure, the angle threshold was selected to be between 270° and 320°, which proved to be the optimal range for accurately describing the crater defect. In the case of undercut defects, the angle threshold is taken to be between 120° and 180°, which provides the most accurate description of the undercut defect.

**Figure 13:**
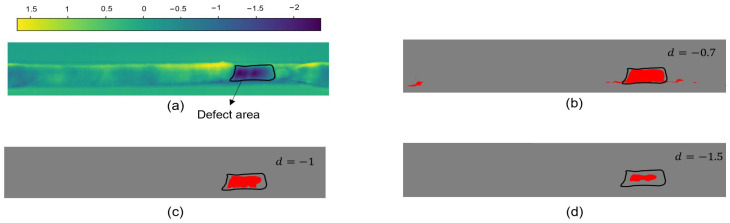
(**a**) Difference results between target and template point clouds; (**b**) extraction results with a height threshold of −0.7; (**c**) extraction results with a height threshold of −1; (**d**) extraction results with a height threshold of −1.5.

The value of the height threshold determines the extraction effect of the point cloud after alignment. Figure 13 shows the extraction result when using different height threshold values: Figure 13a represents the difference between the points of the point cloud after alignment, and we only consider the case where the difference is less than zero. The darker the color, the deeper the defect. In order to avoid false detection, it is necessary to choose an appropriate depth threshold. If the threshold is too low, it will lead to false detection of the weld edge region, as shown in Figure 13b. If the threshold is too high, it will lead to incomplete extraction of the crater region, as shown in Figure 13d. After experimenting, the height threshold was set to −1 for the best extraction of defects.

### 4.4. Defect Detection Results

In order to verify the reliability of the research method in this paper, we conducted experiments on the post-welding reservoir point cloud dataset and analyzed the defect recognition effect and performance. In this paper, 277 different 3D welded point cloud data collected in the actual production are tested, including 100 packages of normal welds and 177 packages of three kinds of defective welds. Over 95% of the defects exhibited a size range between 5 and 500 mm^2^. After preprocessing operations such as planar calibration and downsampling, this paper evaluates the defect detection method based on the accuracy of target defect detection as well as the precision.

The detect result of the algorithm on defects is shown in Table 1. Among the 100 normal weld data, 4 welds were incorrectly detected as defects, and among the 177 defective welds, 2 defects were not detected correctly. As can be seen from the data in the table, the accuracy of the algorithm is 97.1%. The causes of false detection mainly appear in the combination of the arc starting point and the arc closing point of the weld, which has a slight biting phenomenon with the fusion area due to the more obvious height change, which causes some detection interference.

We further validated the ability of the algorithm to classify defects and calculated several metrics to further evaluate the performance of the algorithm, including accuracy and precision.

In 38 cases of hole defects, there were no false detections, but one was missed. In addition, 89 cases of undercut defects had 1 case misdetected as a crater defect. For 50 cases of crater defects, 1 case was falsely detected as a hole and 1 case escaped detection. Overall, the proposed defect detection algorithm can effectively detect and accurately categorize most of the surface defects. The experimental results show that the algorithm performs well in detecting and classifying surface defects, the minimum detectable defect size is 2 mm^2^, and the overall accuracy of defect classification reaches 98.9%.

**Figure 14:**
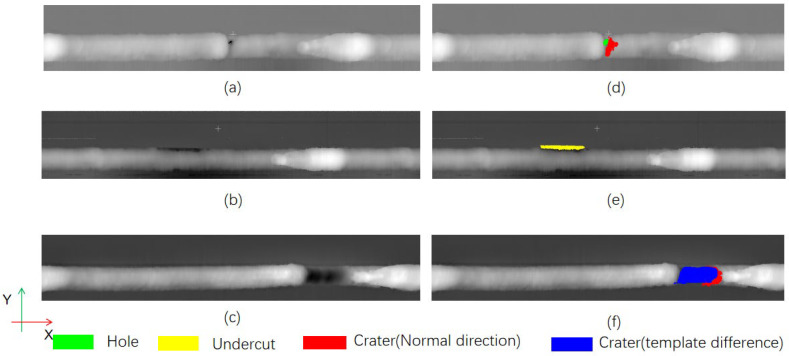
Different types of defects and their extraction results: (**a**) depth map of hole and crater defects; (**b**) hole and crater defect extraction results; (**c**) depth map of undercut defects; (**d**) undercut defect extraction results; (**e**) depth map of crater defects; (**f**) crater defect extraction results.

Finally, we compared the areas of the three defects measured manually with the areas derived by the algorithm. Table 2 shows the results of the comparison, which indicate that the algorithm is able to accurately measure the areas of the different defects. In particular, for crater defects, we compared the detection results of the normal vector discovery detection method alone, the template difference method alone, and the combine of the both methods. The results show that the errors are in the larger range of 80% and 29% when both methods are used alone for detection, while the detection error is reduced to 17% when using a combination of the two methods. The area measured by the algorithm for other types of defects was within 20% of the error of the manually measured area. Some of the extraction results for different defects are also shown in Figure 14, where, for the crater defect, Figure 14f exhibits the extraction results of the two methods, with the blue region indicating the extraction results using template matching, and the red region indicating the extraction results using normal vector direction.

## 5. Conclusions

To provide an effective method for detecting weld defects in aluminum reservoirs of motor vehicles, this paper proposes a 3D vision-based reservoir welding surface defect detection method. The key in this paper is the use of point cloud surface feature information combined with weld defect morphology to realize the extraction and classification of defects. First of all, we use a correction method for contour line’s slope to realize the correction of the surface distortion point cloud data. According to the defect morphology, the defects are extracted using the point cloud surface features. According to the characteristics of the defects in the point cloud data, the classification of the defects is realized using an appropriate threshold value. From the results, the correction effect of the corrected point cloud is significantly improved in this paper compared to the RANSAC method. The accuracy of defect recognition reaches 97.1%, the classification accuracy of defects reaches 98.9%, and the measurement error of the area of different defects is lower than 20%, which can meet the requirements of on-site production. Overall, this method still has its limitations. Firstly, the test object is a specific product, so the method may not be applicable to the detection of other types of welds. Additionally, the experiment was predicated on a limited quantity of data, in particular, a small number of samples with defects such as holes. The limited sample size and diversity may not adequately mirror the conditions typically encountered in practical applications. Additionally, the experiment did not verify the welds of different product models, which could potentially limit the method’s generalizability. To address these limitations, future research will involve the collection of more comprehensive data and the verification of the method’s generalizability across different product models and welding techniques. And although this method achieved good detection results on the existing dataset, in future research, we will continue to try to compare other classification techniques (e.g., using the YOLO method) to further validate the performance of the method and improve it. For future research directions, we plan to collect more comprehensive data and validate the generalizability of the method to welds of different product models and different welding techniques.

## Figures and Tables

**Table 1:** Results of defect detection.

	Normal Weld	Hole Defect	Undercut Defect	Crater Defect	Total
**The number of samples**	100	38	89	50	277
**Proportion to total**	36%	14%	32%	18%	100%
**The number of false detection**	4	0	1	1	6
**The number of missing detection**	0	1	0	1	2
**Accuracy rate**	96%	97.3%	98.8%	96%	97.1%
**Precision**	-	100%	98.8%	98%	98.9%

**Table 2:** Comparison results of manual and algorithmic area measurements.

	Manual Measurement (mm^2^)	Algorithm Measurement (mm^2^)	Relative Error
**Hole**	14.6	15.9	9%
**Undercut**	96.1	78.6	18%
**Crater**	298.3	60.1 (normal direction)	80%
		212.2 (template difference)	29%
		248.7 (combined)	17%

## Data Availability

Data are not publicly available and can be obtained by contacting the corresponding author if necessary.
